# Accumulation of Heavy Metals in Crayfish and Fish from Selected Czech Reservoirs

**DOI:** 10.1155/2014/306103

**Published:** 2014-03-16

**Authors:** Iryna Kuklina, Antonín Kouba, Miloš Buřič, Ivona Horká, Zdeněk Ďuriš, Pavel Kozák

**Affiliations:** ^1^Faculty of Fisheries and Protection of Waters, South Bohemian Research Center of Aquaculture and Biodiversity of Hydrocenoses, University of South Bohemia in Ceske Budejovice, Zátiší 728/II, 389 25 Vodňany, Czech Republic; ^2^Department of Biology and Ecology, Faculty of Science, University of Ostrava, Chittussiho 10, 710 00 Ostrava, Czech Republic

## Abstract

To evaluate the accumulation of aluminium, cadmium, chromium, copper, lead, mercury, nickel, and zinc in crayfish and fish organ tissues, specimens from three drinking water reservoirs (Boskovice, Landštejn, and Nová Říše) and one contaminated site (Darkovské moře) in the Czech Republic were examined. Crayfish hepatopancreas was confirmed to be the primary accumulating site for the majority of metals (Cu > Zn > Ni > Cd > Cr), while Hg and Cr were concentrated in abdominal muscle, and Al and Pb were concentrated in gill. Metals found in Nová Říše specimens included Cu > Zn > Ni and those found in Boskovice included Zn > Hg > Cr. Cd concentrations were observed only in Landštejn specimens, while contaminated Darkovské moře specimens showed the highest levels of accumulation (Cu > Al > Zn > Pb). The majority of evaluated metals were found in higher concentrations in crayfish: Cu > Al > Zn > Ni > Cr > Cd > Pb, with Hg being the only metal accumulating higher in fish. Due to accumulation similarities of Al in crayfish and fish gill, differences of Hg in muscle, and features noted for the remaining metals in examined tissues, biomonitoring should incorporate both crayfish and fish to produce more relevant water quality surveys.

## 1. Introduction

Maintaining suitable freshwater quality is essential for both aquatic and terrestrial life. Monitoring based on relevant bioindicators provides useful data for evaluation of environmental status [1]. Although the hazards of water contamination by heavy metals are well known, it remains an issue due to expanding industrial development, including mining activities [2]. Macroinvertebrates are frequently suggested as bioindicators for monitoring changing water conditions in areas of potential contamination [3]. In practice, crayfish are of particular importance for biomonitoring studies [4], being the keystone species in most ecosystems in which they occur [5], and, most importantly, can tolerate polluted environments and reflect pollution levels due to accumulation of respective elements in their tissues [6]. Algae and fish are also successfully employed in biomonitoring programmes, although algae can sometimes be difficult to identify, while fish are mobile and can potentially avoid contaminated areas [3]. Water body contamination can be assessed by the quantity of selected elements accumulated in target organisms and their tissues. Much study had been devoted to the assessment of heavy metal bioaccumulation in aquatic biota [2], including crayfish [7] and fish [8], with each bioindicator having its merits [9]. Quantification of bioaccumulation of hazardous chemicals as indicated by their concentrations in organ tissue is the basis of biomonitoring [10]. While crayfish are useful as bioindicators of contamination, they are also a valuable food source [11], making monitoring of organ tissue metal concentrations relevant to both animal and human health.

This primary objective of this study was to survey metal concentrations in crayfish as representative biota of drinking water reservoirs and to relate these results to data on metal accumulation in fish from the same areas. The establishment of such relation was important for underlining specific attributes of selected elements accumulation for examined reservoirs and resident species.

## 2. Materials and Methods

### 2.1. Studied Localities

Heavy metal content in organ tissue was assessed in crayfish from three Czech water supply reservoirs: Boskovice (South Moravian Region; 49°29′50′′N, 16°41′59′′E), Landštejn (South Bohemian region; 49°1′21′′N, 15°14′30′′E), and Nová Říše (Vysočina region; 49°29′50′′N, 16°41′59′′E). A fourth reservoir known to be contaminated with heavy metals, Darkovské moře (Moravian-Silesian Region; 49°49′56.935′′N, 18°33′10.230′′E), was used as contaminated. The contaminated reservoir is a lowland (maximum surface area 32 ha, depth 28 m) flooded by ground waters in the 1990s, located in a region highly affected by coal mining. The shoreline and vicinity are formed by gangue deposits. The reservoir is currently used for recreation.

### 2.2. Crayfish Sampling

Crayfish were caught in baited traps from June to November 2008: Boskovice on 10 June, Nová Říše on 19 June, Landštejn on 19 June and 9 July, and Darkovské moře on 7 and 12 November. For each site, 10 intermolt males were selected from trapped noble crayfish,* Astacus astacus* (L. 1758). Crayfish were grouped based on carapace length (CL) and the postorbital carapace length (POCL) to the nearest 0.1 mm and total weight (TW) to 0.1 g. Crayfish measurements from test sites were CL = 48.4 ± 1.9 mm, POCL = 36.5 ± 1.6 mm, and TW = 28.7 ± 3.3 g, and for contaminated locality: CL = 59.9 ± 2.2 mm, POCL = 41.9 ± 1.7 mm, and TW = 39.5 ± 6.7 g. The crayfish from the Darkovské moře were significantly larger than those from drinking water reservoirs, where crayfish size did not significantly differ.

### 2.3. Metals Analysis

Prior to dissection, the selected crayfish were immediately immersed in liquid nitrogen or, for specimens from the control site, subjected to short-term freezing, and samples of abdominal muscle, hepatopancreas, and gill were obtained. Abdominal muscle and hepatopancreas were analysed for zinc, cadmium, lead, copper, nickel, chromium, and mercury content. In the crayfish specimens from Darkovské moře, nickel was not measured, and hepatopancreas, abdominal muscle, and gill were analysed for zinc, cadmium, lead, copper, chromium, and mercury. Gills of crayfish from all sites were also analysed for the presence of aluminium. Because of the small amount of gill tissue available for aluminium analyses, tissue samples from two randomly selected individuals were paired into five samples for each test locality, while gill samples of crayfish from Darkovské moře were analysed individually.

Metal analyses were carried out in accredited laboratories of State Enterprise Povodí Moravy (Brno, Czech Republic) using operating procedures according to EN International Organization for Standardization (ISO) standards.

Al, Cd, Cr, Cu, Pb, Ni, and Zn determination was performed according to the method DIN EN ISO 17294-2:2005 (water quality, application of inductively coupled plasma mass spectrometry, ICP-MS) using the inductively coupled plasma mass spectrometer Perkin Elmer Elan DRC-e (Perkin Elmer, Waltham, MA, USA). Detection limits were as follows: 5 mg kg^−1^ dry weight for Zn and Al, 0.5 mg kg^−1^ for Cr, Cu, Pb, and Ni, and 0.05 mg kg^−1^ for Cd. The samples were freeze-dried using Christ Alpha 1-2 lyophilizer, grinded in Retsch spherical mill, and decomposed with Milestone Ethos-1 microwave decomposition apparatus (Czech technical standards ČSN EN 13657, solid matrices samples preparation, screening, and skeleton determination).

Samples for Hg determination were processed according to the Czech technical norms, ČSN 757440 (determination of total mercury by atomic absorption spectroscopy), on AMA-254 Analyzer (Altec, Prague, Czech Republic) by direct measurement, without microwave decomposition, with detection limit of 0.01 mg kg^−1^. Metals concentrations are expressed on the dry weight (dw) basis.

### 2.4. Metal Detection in Fish

State Enterprise Povodí Moravy provided data on Zn, Cd, Pb, Cu, Hg, and Al in muscle of the common bream* Abramis brama* (L., 1758), common rudd* Scardinius erythrophthalmus *(L., 1758), European perch* Perca fluviatilis *(L., 1758), pikeperch* Sander lucioperca *(L., 1758), roach* Rutilus rutilus *(L., 1758), and tench* Tinca tinca* (L., 1758) of different size and age (Table 1). Analytical methods were similar to those used for crayfish. The assessment was conducted in the same sites, with the exception of Darkovské moře, at approximately the same time period in 2008: Nová Říše, 19 June; Boskovice, 9 July; Landštejn, 30 September.

### 2.5. Statistical Analyses

Crayfish biometric parameters were tested for data normality (Kolmogorov-Smirnov test) and homoscedasticity (Levene test) and compared among sites by nonparametric Kruskal-Wallis test (Table 2), because of heterogeneity of variances.

For statistical analysis, metal concentrations below the detection limit were replaced with the mentioned detection limits. Differences in metal content of crayfish tissue among localities were evaluated using the nonparametric Kruskal-Wallis test (Tables 3 and 4), followed by multiple means comparison of all groups as a post hoc test.

To evaluate whether the measured metals showed greater accumulation in hepatopancreas or abdominal muscle of crayfish, the nonparametric Wilcoxon test for matched pairs was conducted (Table 5). To compare accumulation in abdominal muscle, hepatopancreas, and gill of specimens from the contaminated site, the Friedman ANOVA test was applied (Table 6). Aluminium accumulation in crayfish gill from all localities was evaluated using the Kruskal-Wallis test (Table 7) followed by multiple comparisons of mean ranks for all groups.

The significance level for all tests was ≤0.05, while statistical evaluation was conducted using STATISTICA.10 software for Windows (StatSoft, Czech Republic). Data are presented as mean values ± standard deviations.

Biometric parameters of fish and metal concentration in fish muscle were not statistically compared due to variation in age and size of specimens.

## 3. Results

### 3.1. Selected Metals Content in Crayfish Hepatopancreas

Zinc content was significantly lower in the specimens from the Landštejn Reservoir, 100.29 ± 34.98 mg kg^−1^, with concentrations in specimens from other reservoirs nearly double that value (Table 8). Landštejn specimens showed the highest Cd content, 7.31 ± 2.56 mg kg^−1^ (Table 8). Pb content was below the detection limit (<0.50 mg kg^−1^) for all sites except the Landštejn Reservoir at 0.82 ± 0.35 mg kg^−1^. The lowest Cu content was detected in samples from Landštejn, 30.41 ± 32.22 mg kg^−1^, with a higher concentration in the Nová Říše Reservoir (410.10 ± 154.70 mg kg^−1^) and significantly higher concentration at the contaminated site (794.70 ± 234.74 mg kg^−1^). The highest concentration of Ni, 13.72 ± 9.99 mg kg^−1^, was found in crayfish from the Nová Říše Reservoir, while it was not detected in those from Darkovské moře. The highest Cr content was in Boskovice and Landštejn Reservoirs at 3.76 ± 1.57 and 2.49 ± 2.63 mg kg^−1^, respectively. Hg content was 0.14 ± 0.09 mg kg^−1^ in Boskovice, while it was half that level in Darkovské moře, at 0.07 ± 0.03 mg kg^−1^.

### 3.2. Selected Metal Content in Crayfish Abdominal Muscle

There was no significant difference in Zn content of crayfish abdominal muscle among drinking water reservoirs (Table 9), but approximately double its mean concentration was observed in the contaminated site (128.23 ± 44.33 mg kg^−1^). Cd content was below the detection limit in drinking water reservoirs but 0.13 ± 0.08 mg kg^−1^ at the contaminated site. Pb was below the detection limit at all sites. The specimens from the contaminated reservoir showed highest Cu content (55.97 ± 14.07 mg kg^−1^), while the lowest Cu concentration, 20.92 ± 4.34 mg kg^−1^, was found in Landštejn. No difference was found in either Ni or Cr content among the sampled areas. The highest Hg concentration was seen in Boskovice Reservoir (1.18 ± 0.31 mg kg^−1^) (Table 9).

### 3.3. Aluminium Content in Crayfish Gill

Aluminium content of crayfish gill did not significantly differed among water storage reservoirs (50 ± 10–170 ± 130 mg kg^−1^), while samples from the contaminated locality had significantly higher levels, 780 ± 700 mg kg^−1^ (Table 10).

### 3.4. Target Tissue of Metal Accumulation in Crayfish

**Figure 1:**
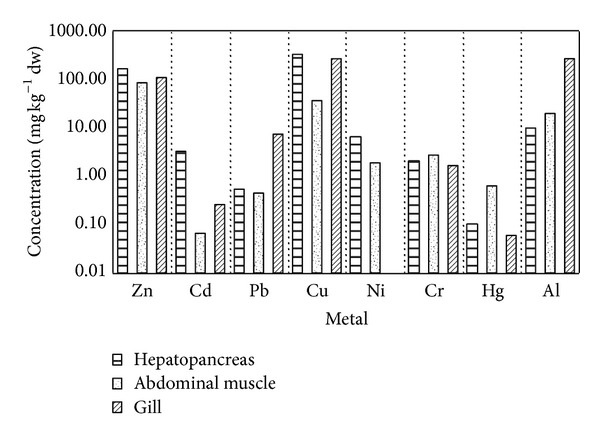
Concentration of metals (zinc, cadmium, lead, copper, nickel, chromium, mercury) in crayfish organ tissues (*n* = 10, for each tissue): hepatopancreas, abdominal muscle, gill. Averages of combined locality data are presented. An ordinate is presented in logarithmic scale.

Analysis across all sampling sites indicated that the crayfish digestive organ (hepatopancreas) was the primary accumulation site of the majority of studied metals. This was noted for Zn, Cd, and, to some extent, for Cu and Ni (Figure 1). Cr was found in both hepatopancreas and abdominal muscle but was higher in the latter (Figure 1). Hg primarily accumulated in crayfish abdominal muscle at similar levels for all sites (Tables 8 and 9). Pb and Al mainly accumulated in crayfish gill (Figure 1).

### 3.5. Reservoir Comparisons

**Figure 2:**
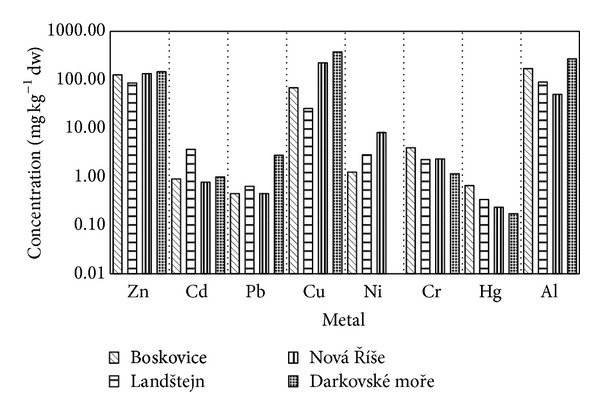
Concentration of metals (zinc, cadmium, lead, copper, nickel, chromium, mercury) in crayfish (*n* = 10, for each site) from selected localities (Boskovice, Landštejn, Nová Říše, Darkovské moře). Averages of combined data, abdominal muscle, hepatopancreas, and gill are presented. An ordinate is presented in logarithmic scale.

The highest levels of Cd were found in crayfish from Landštejn, while they were the lowest in content of other analysed metals. Darkovské moře showed the highest concentrations of Zn, Pb, Cu, and Al. Nová Říše crayfish had high Zn, Cu, and Ni concentrations. Similar to Darkovské moře and Nová Říše, Boskovice samples showed high Zn content, as well as high Cr and Hg concentrations (Figure 2).

### 3.6. Selected Metal Content in Fish and Crayfish from Drinking Water Reservoirs

**Figure 3:**
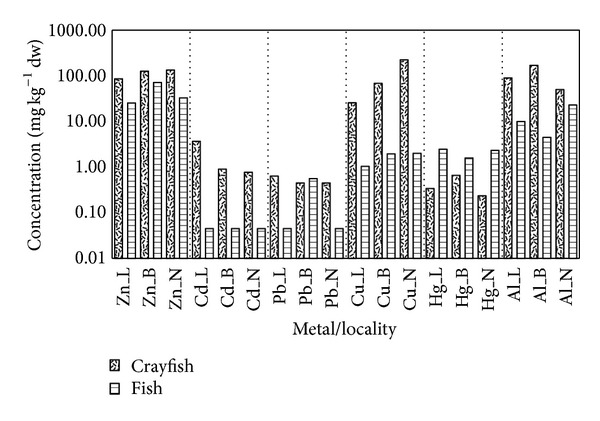
Concentration of metals (zinc, cadmium, lead, copper, mercury, and aluminium) in crayfish tissue (abdominal muscle, hepatopancreas, gill) and fish muscle from selected water storage reservoirs: Boskovice (B, crayfish *n* = 10, fish *n* = 6), Landštejn (L, crayfish *n* = 10, fish *n* = 5), Nová Říše (N, crayfish *n* = 10, fish *n* = 5). An ordinate is presented in logarithmic scale.

Metal accumulation in crayfish hepatopancreas, abdominal muscle, and gill tissue and in fish muscle tissues (Table 11) was compared among drinking water reservoirs. In general, metal concentrations were significantly higher in crayfish (Figure 3). The only metal occurring in higher amounts in fish muscle compared to crayfish was Hg (2.10 ± 1.77 mg kg^−1^ versus 0.41 ± 0.42 mg kg^−1^), while Pb was found in similar amounts (0.55 ± 0.24 mg kg^−1^ for fish and 0.57 ± 0.21 mg kg^−1^ for crayfish), although it tended to be higher in crayfish. Fish from the Boskovice Reservoir had the highest Zn content (71.50 ± 34.60 mg kg^−1^) and the lowest Hg concentration (1.59 ± 0.53 mg kg^−1^). Fish from the Landštejn and Nová Říše reservoirs showed similar levels of Zn (25.42 ± 9.57 mg kg^−1^ and 32.90 ± 5.88 mg kg^−1^, resp.). Both fish and crayfish from Landštejn contained the lowest amounts of Cu (1.04 ± 0.32 mg kg^−1^ and 25.67 ± 6.71 mg kg^−1^, resp.), while the highest Hg concentration in fish (2.47 ± 2.73 mg kg^−1^) was detected at that site. The lowest Al concentration in fish was in Boskovice Reservoir, where levels were below the detection limit.

### 3.7. Zinc, Cadmium, Lead, Copper, and Mercury in Fish Muscle Compared with Crayfish Abdominal Muscle

**Figure 4:**
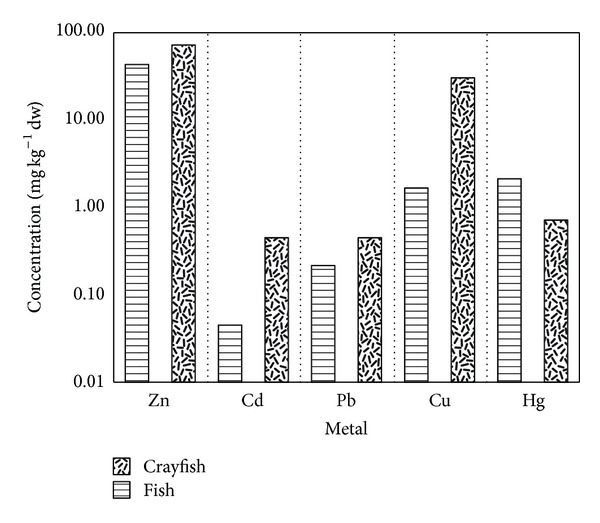
Concentration of selected metals (zinc, cadmium, lead, copper, mercury) in crayfish (*n* = 30) abdominal muscle and fish (*n* = 16) muscle throughout water reservoirs (means of combined data Boskovice, Landštejn, Nová Říše). An ordinate is presented in logarithmic scale.

Zinc and Cu content was significantly lower in fish muscle compared with crayfish abdominal muscle, while Pb was near the detection limit in both fish and crayfish (0.55 ± 0.24 mg kg^−1^ and 0.50 mg kg^−1^, resp.), and Cd was below the detection level, in fish. Hg in fish (perch, 4.00 ± 1.88 mg kg^−1^, > pikeperch, 2.33 mg kg^−1^, > rudd, 2.11 ± 0.30 mg kg^−1^, > tench, 1.15 ± 0.65 mg kg^−1^, > roach, 0.83 ± 0.23 mg kg^−1^, > bream, 0.62 ± 0.68 mg kg^−1^) was detected in higher amounts than in crayfish, 0.72 ± 0.40 mg kg^−1^, (Figure 4).

## 4. Discussion

As expected, hepatopancreas of crayfish showed the highest accumulation rate for the majority of evaluated metals. Thus, when the goal is to obtain relevant content of Zn, Cd, Cu, or Ni in crayfish as biomonitors it is advisable to assess levels in hepatopancreas. Analysing other tissues for these metals may result in concentrations appearing low or remaining undetected.

Cr can be detected in hepatopancreas and abdominal muscle in relatively equal amounts. Although Cr is toxic to aquatic organisms [12], it is not included as potentially hazardous to humans by the European Commission (EC) Regulation [13] setting maximum levels for foods. Jorhem et al. [11] reported that Cr concentrations in hepatopancreas of the noble crayfish rose several-fold after boiling. Jorhem et al. [11] and Mackevičienė [14] found Cr concentrations in abdominal muscle of noble crayfish caught in unpolluted Swedish and Lithuanian lakes to be 0.13 and 0.30 mg kg^−1^, respectively, and in hepatopancreas, 0.15 and 0.25 mg kg^−1^, respectively, compared to our findings of 2.03–4.19 mg kg^−1^ for abdominal muscle and 0.87–3.76 mg kg^−1^ for hepatopancreas. The lowest concentrations of Cr were found in Darkovské moře, which was regarded a contaminated site. Tunca et al. [15], reported similar Cr concentrations in hepatopancreas (0.65 mg kg^−1^) and abdomen (0.50 mg kg^−1^) of the narrow-clawed crayfish from a Turkish lake, collected in the same season as our study, at an unpolluted site. Tunca et al. [16] reported Cr concentration in gill to be somewhat higher than in our study (5.3 mg kg^−1^ versus 1.67 mg kg^−1^), as was Ni, which was not detected in the crayfish from Darkovské moře. Although Tunca et al. [16] found a positive Cr/Cu correlation (*r* = 0.53) that could explain some trends of Cr accumulation in crayfish tissue, we found no relationship between these metals in our survey.

Zn, Cu, and Ni, which commonly accumulate mainly in hepatopancreas of other crayfish species as well [7], were found to be substantially decreasing after cooking [11]. However, concentrations of Zn and Cu in abdominal muscle have been shown to slightly increase after boiling [11]. Zn, Ni, and Cu are also not considered potentially harmful to humans according to EC regulations [13]. From an animal welfare point of view, Zn and Ni concentrations in our survey were in agreement with reviews of Eisler [17, 18] and results of Jorhem et al. [11] and Mackevičienė [14], who reported Ni levels (0.50–0.85 mg kg^−1^) similar to our observations (1.38–2.55 mg kg^−1^) in abdominal muscle and 1.54–3.54 mg kg^−1^ compared with our findings (1.11–13.72 mg kg^−1^) in hepatopancreas. Zinc and Cu in abdominal muscle (23.25–75.00 mg kg^−1^ versus 68.60–128.23 mg kg^−1^ and 6.10–28.50 mg kg^−1^ versus 20.92–55.97 mg kg^−1^, resp.) and Cu in hepatopancreas (4.93–185.71 mg kg^−1^ versus 30.41–794.70 mg kg^−1^) were found in higher concentrations in specimens from the Czech reservoirs than in those from Swedish and Lithuanian waters [11, 14]. Reported differences can largely depend on differing geological characteristics of localities, including environmental concentrations of metals in reservoirs. The highest concentrations of these metals in our study were found in the contaminated site, but not in drinking water basins. This may be linked to mining activity near the contaminated location. In agreement with Bagatto and Alikhan [19, 20], we also observed hepatopancreas to be the major crayfish organ for metal accumulation. However, Tunca et al. [15] reported Cr, Ni, and Cu to accumulate in crayfish gill at greater levels than in hepatopancreas.

When metal content was averaged for all tested crayfish tissues and compared with those in fish, we found no obvious similarities in levels or distribution. In general, metal concentrations were higher in crayfish than in fish. When comparing crayfish abdominal muscle to that of fish muscle, we did not find relationships among concentrations of metals. The majority of analysed elements (Zn, Cd, Pb, and Cu) appeared at higher concentrations in crayfish than in fish (Figure 4). The only element with higher concentration in fish than in crayfish was Hg. Fish data in this study included species feeding on benthic invertebrates (tench), on plankton or benthic invertebrates (roach, bream), and on zooplankton or algae (rudd), as well as predatory fish (pikeperch and perch). It is not surprising that the highest Hg concentrations were found in pikeperch, 3.54–6.13 mg kg^−1^, and perch, 1.06–2.33 mg kg^−1^, since these species occupy a higher trophic level [21]. Since Hg biomagnifies through the food web [22, 23], Hg content in fish would be expected to exceed that in crayfish. If crayfish prey upon other benthic invertebrates, Hg biomagnification would be a factor in those species also [24].

The highest Hg levels were found in crayfish from the Boskovice Reservoir, which did not appear to be the most contaminated among the studied sites with respect to other metals. We cannot suggest that Boskovice is the site polluted by Hg because of its highest amounts there, as this metal is actively transported through the trophic web [25], particularly in the initial years of reservoir exploitation. In this connection, Boskovice Reservoir was the youngest, constructed in 1990, while Nová Říše and Landštejn reservoirs have been in use since 1985 and 1973, respectively. There is no information on Hg content in crayfish abdominal muscle in EC Regulations [13], but 1.00 mg kg^−1^ of Hg in muscle of fresh fish is within safe limits for the human health and for aquatic animal welfare [26]. Our data for the omnivorous fish, bream and roach, are in agreement with Noël et al. [8], who looked at fish from uncontaminated sites, but Hg content for predatory perch (0.47 mg kg^−1^) and pikeperch (0.94 mg kg^−1^) was several times higher in our study (4.00 mg kg^−1^ and 2.33 mg kg^−1^, resp.). However, we analysed larger fish, perch of average 434 g compared to 105 g and pikeperch of average 3300 g compared to 1002 g. Červenka et al. [27], who analysed fish muscle from fresh water reservoirs, observed similarly high, 6.41 mg kg^−1^, Hg levels in perch, but higher levels in bream, 2.78 mg kg^−1^ compared with 0.64 mg kg^−1^ in the present study. Svobodova et al. [28] found less than 0.05 mg kg^−1^ (fresh weight) Hg in common carp* Cyprinus carpio* (L. 1758); however, with respect to Cd, Pb, and Cu our results are in agreement. Cd and Cu in fish also did not exceed values established by EC Regulations [13] and Eisler [29, 30]. The noble crayfish is regarded as an Hg bioindicator by Loukola-Ruskeeniemi et al. [31], who detected concentrations in crayfish abdominal muscle (0.88 mg kg^−1^) similar to that of the present study (0.39–1.18 mg kg^−1^) in Finland, while we found less Hg in hepatopancreas, 0.07–0.14 mg kg^−1^, compared with 0.24 mg kg^−1^ reported for Finnish crayfish.

We analysed crayfish gill for presence of Al, as its concentration could serve as a reference value for investigation of impact on crayfish of aluminium-containing compounds employed for water treatment. The common coagulation agent PAX-18 is such a compound, as it contains polyaluminium chloride (9% aluminium) as an active ingredient and is widely used to precipitate orthophosphates, which cause water eutrophication, and to reduce phytoplankton bloom [32]. However, together with phytoplankton control, polyaluminium chloride can cause harmful effects in nontarget aquatic organisms, especially those most vulnerable to the impact and that easily accumulate aluminium, primarily juvenile fish [32] and crustaceans [33], but also other benthic organisms.

It is difficult to assess the level of toxicity of the observed aluminium, and whether the levels found in crayfish gill are the result of metal pollution. The high, 780 mg kg^−1^, aluminium level in gill of crayfish from the contaminated site presumes its contamination. Similar Al levels were reported by Madigosky et al. [34], who found up to 981 mg kg^−1^ in gill of the red swamp crayfish,* Procambarus clarkii* (Girard, 1852), from road-side ditches along highways in northern Louisiana, USA. While we primarily focused on drinking water reservoirs, it is necessary to consider Darkovské moře. Al concentrations in hepatopancreas at 10 mg kg^−1^ and abdominal muscle at 20 mg kg^−1^ were found in crayfish from the site. Alexopoulos et al. [35], after 20-day exposure to Al at 500 *μ*g L^−1^, found approximately 1200 mg kg^−1^ in signal crayfish* Pacifastacus leniusculus* (Dana, 1852) gill, 10 mg kg^−1^ in abdominal muscle, and 20 mg kg^−1^ in the digestive gland. Gill concentration measured in our study would correspond approximately to 14 days of such exposure, while in abdominal muscle and hepatopancreas, Al levels would be half that value, but still of a similar effect. According to Macova et al. [32], amounts of PAX-18 commonly used for treatment of natural waters, 45–90 mg kg^−1^ equivalent to 5–10 mg kg^−1^ of Al, are safe for common carp juveniles. The treatment dose in Alexopoulos et al. [35] was 10% of that commonly used. Therefore, we can suppose that Al content in crayfish gill from the Czech drinking water reservoirs, 50–170 mg kg^−1^, was not evidence of contamination, as these concentrations were much lower than those found in contaminated site. As in crayfish, gill is the target for Al uptake in fish [36, 37], since Al binds to the gill due to the mucus secreted by these organs that causes their damage and mucus intensive buildup [38, 39]. Fish muscle showed lower, 4.5–23.0 mg kg^−1^, Al concentrations compared to those reported by Coetzee et al. [40], 11–109 mg kg^−1^, and 22.5–40.6 mg kg^−1^ found by Sapozhnikova et al. [41]. Although the highest Al concentrations are usually accumulated in gill, we agree with Coetzee et al. [40] that, in monitoring, muscle should also be considered.

## 5. Conclusions

Various aquatic organisms should be used in biomonitoring studies to give a more complete picture of environmental pollution. Crayfish, due to low migration across water bodies, may provide more precise data than do fish. In biomonitoring, some potentially toxic elements, such as Al and Hg, can be over- or underestimated, depending on considered tissue and species and the stage of their life cycle. Thus either crayfish abdominal or fish muscle for Al bioaccumulation assessment is recommended, while for Hg surveys, fish, especially carnivorous species, should not be used because of their potential for biomagnification. The remaining metals, Cd, Cu, Ni, and Zn, except of Cr and Pb, are primarily accumulated in crayfish hepatopancreas, making this tissue the recommended target for bioaccumulation studies. Expansion of data sources, species, tissues, and sampling sites will produce more relevant biomonitoring surveys. The last is highly important, not just for environmental preservation, but for evaluation of the potential effects on human health.

## Figures and Tables

**Table 1:** Biometric parameters (TL: total length, *W*: weight) of fish (*n* = 16) from drinking water reservoirs.

Reservoir/Species	TL, (mm)	*W*, (g)	Age, (yr)
Boskovice			
*Abramis brama *	350	400	4
*Scardinius erythrophthalmus *	230	120	6
*Scardinius erythrophthalmus *	220	100	5
*Perca fluviatilis *	190	90	4
*Rutilus rutilus *	270	190	5
*Tinca tinca *	440	850	5
Landštejn			
*Abramis brama *	510	1600	8
*Perca fluviatilis *	280	250	5
*Perca fluviatilis *	330	400	7
*Rutilus rutilus *	330	380	5
*Tinca tinca *	450	1100	11
Nová Říše			
*Abramis brama *	400	670	5
*Perca fluviatilis *	330	450	6
*Perca fluviatilis *	380	980	9
*Sander lucioperca *	710	3300	7
*Tinca tinca *	400	850	5

**Table 2:** Statistical evaluation of crayfish biometric parameters.

Test	CL	POCL	TW
*KW* − *H* (3, 40)	22.79	22.08	18.02
*P*	<0.05	<0.05	<0.05

**Table 3:** Statistical comparison of metal accumulation in hepatopancreas of crayfish from Boskovice, Landštejn, Nová Říše, and Darkovské moře.

Test	Cd	Cr	Cu	Pb	Hg	Ni	Zn
*KW* − *H* (3, 40)	25.81	25.46	32.57	34.39	12.87	5.61*	15.54
*P*	<0.05	<0.05	<0.05	<0.05	<0.05	>0.05	<0.05

*df = 2 since nickel was not detected in crayfish hepatopancreas from Darkovské moře.

**Table 4:** Statistical comparison of metal accumulation in abdominal muscle of crayfish from Boskovice, Landštejn, Nová Říše, and Darkovské moře.

Test	Cd	Cr	Cu	Pb	Hg	Ni	Zn
*KW* − *H* (3, 40)	33.07	24.36	27.96	39.00	27.35	6.54*	23.55
*P*	<0.05	<0.05	<0.05	<0.05	<0.05	<0.05	<0.05

*df = 2 since nickel was not detected in crayfish abdominal muscle from Darkovské moře.

**Table 5:** Statistical comparison of metal accumulation in crayfish hepatopancreas and abdominal muscle among drinking water reservoirs.

Locality/Test	Cd	Cr	Cu	Pb	Hg	Ni	Zn
Boskovice							
*W*-*Z* (1, 10)	2.70	2.60	2.29	2.80	2.80	1.75	2.80
*P*	<0.05	<0.05	<0.05	<0.05	<0.05	>0.05	<0.05
Landštejn							
*W*-*Z* (1, 10)	2.80	0.06	0.66	2.37	2.80	2.31	2.29
*P*	<0.05	>0.05	>0.05	<0.05	<0.05	<0.05	<0.05
Nová Říše							
*W*-*Z* (1, 10)	2.80	2.70	2.80	2.80	2.80	2.50	2.80
*P*	<0.05	<0.05	<0.05	<0.05	<0.05	<0.05	<0.05

**Table 6:** Statistical comparison of metal accumulation in crayfish hepatopancreas, abdominal muscle, and gill for Darkovské moře Reservoir.

Test	Al	Cd	Cr	Cu	Pb	Hg	Zn
*F* (2, 10)	16.00	15.62	10.57	20.00	20.00	15.44	16.80
*P*	<0.05	<0.05	<0.05	<0.05	<0.05	<0.05	<0.05

**Table 7:** Statistical evaluation of aluminium accumulation in gill of crayfish from Boskovice, Landštejn, Nová Říše, and Darkovské moře.

Test	Al
*KW* − *H* (3, 25)	18.68
*P*	<0.05

*n* = 25 since gill of crayfish from Boskovice, Landštejn, and Nová Říše were pooled into 5 samples for each locality.

**Table 8:** Metal concentration (mg kg^−1^ dw) in crayfish (*n* = 10 from each locality) hepatopancreas. Values are given as mean ± s.d.

Locality	Aluminium	Cadmium	Chromium	Copper	Lead	Mercury	Nickel	Zinc
Boskovice	N/D	1.76 ± 0.43^b^	3.76 ± 1.57^a^	103.97 ± 105.37^bc^	<0.50^b^	0.14 ± 0.09^a^	1.11 ± 1.33^b^	176.10 ± 56.22^a^
Landštejn	N/D	7.31 ± 2.56^a^	2.49 ± 2.63^ab^	30.41 ± 32.22^c^	0.82 ± 0.35^a^	0.10 ± 0.03^ab^	3.89 ± 2.60^ab^	100.29 ± 34.98^b^
Nová Říše	N/D	1.50 ± 0.40^b^	0.87 ± 1.08^b^	410.10 ± 154.70^ab^	<0.50^b^	0.08 ± 0.04^ab^	13.72 ± 9.99^a^	199.60 ± 59.80^a^
Darkovské moře	10 ± 10	2.58 ± 1.36^b^	0.80 ± 0.67^b^	794.70 ± 234.74^a^	<0.50^b^	0.07 ± 0.03^b^	N/D	200.10 ± 58.46^a^

^a,b,c^Values marked by different letters differed significantly at *α* < 0.05.

N/D Metal was not detected.

**Table 9:** Metal concentration (mg kg^−1^ dw) in crayfish (*n* = 10 from each locality) abdominal muscle. Values are given as mean ± s.d.

Locality	Aluminium	Cadmium	Chromium	Copper	Lead	Mercury	Nickel	Zinc
Boskovice	N/D	<0.05^b^	4.19 ± 6.82^a^	32.89 ± 7.94^bc^	<0.50^a^	1.18 ± 0.31^a^	1.46 ± 1.38^a^	76.87 ± 11.01^b^
Landštejn	N/D	<0.05^b^	2.03 ± 1.00^a^	20.92 ± 4.34^c^	<0.50^a^	0.58 ± 0.06^ab^	1.77 ± 3.40^a^	71.98 ± 7.98^b^
Nová Říše	N/D	<0.05^b^	3.79 ± 5.94^a^	37.60 ± 9.00^ab^	<0.50^a^	0.39 ± 0.15^b^	2.58 ± 6.24^a^	68.60 ± 4.57^b^
Darkovské moře	20 ± 30	0.13 ± 0.08^a^	0.99 ± 0.84^a^	55.97 ± 14.07^a^	<0.50^a^	0.39 ± 0.16^b^	N/D	128.23 ± 44.33^a^

^a,b,c^Values marked by different letters differed significantly at *α* < 0.05.

N/D Metal was not detected.

**Table 10:** Aluminium concentration (mg kg^−1^ dw) in crayfish (*n* = 10 from each locality) gill. Values are given as mean ± s.d.

Locality	Boskovice	Landštejn	Nová Říše	Darkovské moře
Aluminium	170 ± 130^ab^	90 ± 50^b^	50 ± 10^b^	780 ± 700^a^

^a,b^Values marked by different letters differed significantly at *α* < 0.05.

**Table 11:** Metal concentration (mg kg^−1^ dw) in muscle of fish (*n* = 16) from drinking water reservoirs.

Reservoir/Species	Aluminium	Cadmium	Copper	Lead	Mercury	Zinc
Boskovice						
*Abramis brama *	<5.0	<0.05	1.28	<0.5	1.41	33.3
*Scardinius erythrophthalmus *	<5.0	<0.05	2.95	<0.5	2.32	126.0
*Scardinius erythrophthalmus *	<5.0	<0.05	1.55	<0.5	1.90	69.8
*Perca fluviatilis *	<5.0	<0.05	1.55	0.62	1.06	97.3
*Rutilus rutilus *	<5.0	<0.05	1.92	<0.5	0.99	45.3
*Tinca tinca *	<5.0	<0.05	2.42	0.51	1.88	57.3
Landštejn						
*Abramis brama *	6.00	<0.05	<0.5	1.01	0.24	41.8
*Perca fluviatilis *	16.00	<0.05	<0.5	1.16	4.66	23.9
*Perca fluviatilis *	11.00	<0.05	0.93	0.93	6.13	16.9
*Rutilus rutilus *	7.00	<0.05	<0.5	1.50	0.66	23.5
*Tinca tinca *	<5.00	<0.05	<0.5	0.62	0.64	21.0
Nová Říše						
*Abramis brama *	<5.0	<0.05	<0.5	1.34	0.21	38.5
*Perca fluviatilis *	<5.0	<0.05	<0.5	5.33	3.57	32.7
*Perca fluviatilis *	<5.0	<0.05	<0.5	1.03	4.59	38.8
*Sander lucioperca *	<5.0	<0.05	<0.5	0.66	2.33	25.2
*Tinca tinca *	23.00	<0.05	1.33	1.69	0.93	29.3
